# Isolation and identification of antifungal, antibacterial and nematocide agents from marine *bacillus gottheilii* MSB1

**DOI:** 10.1186/s12896-024-00920-y

**Published:** 2024-11-13

**Authors:** Ahmed. S. Shehata, Marwa A. Samy, Sherien E. Sobhy, Aida M. Farag, Ibrahim M. El-Sherbiny, Ahmed A. Saleh, Elsayed E. Hafez, Mamdouh Abdel-Mogib, Haiam M. Aboul-Ela

**Affiliations:** 1 Marine Biotechnology and Natural Product Lab., Environment Division, National Institute of Oceanography and Fisheries, NIOF, Alexandria City, Egypt; 2 Green Materials Technology Department, Environment and Natural Materials Research Institute (ENMRI), City of Scientific Research and Technological Applications (SRTA), New Borg El-Arab City, Alexandria, 21934 Egypt; 3 Plant Protection and Biomolecular Diagnosis Department, Arid Lands Cultivation Research Institute, City of Scientific Research and Technological Applications, New Borg El-Arab City, Alexandria, 21934 Egypt; 4 Department of Nanomaterial Science, Zewil City, Egypt; 5 Animal and Fish Production Department, Faculty of Agriculture (Al-Shatby), Alexandria University, Alexandria City, 11865 Egypt; 6 Chemistry Department, Faculty of Science, Mansoura University, Mansoura, 35316 Egypt; 7 College of Fisheries and Aquaculture Technology, Arab Academy for Science, Technology and Maritime Transport, Abu Qir, Alexandria, Egypt

**Keywords:** Endosymbiotic bacteria, Biopesticide, Antifungal agents, *Bacillus gottheilii* MSB1, Nematocide agents

## Abstract

Pathogenic fungi employ numerous strategies to colonize plants, infect them, reduce crop yield and quality, and cause significant losses in agricultural production. The increasing use of chemical pesticides has led to various ecological and environmental issues, including the emergence of resistant weeds, soil compaction, and water pollution, all negatively impacting agricultural sustainability. Additionally, the extensive development of synthetic fungicides has adverse effects on animal and human health, prompting the exploration of alternative approaches and green strategies for phytopathogen control. Microorganisms living in sponges represent a promising source of novel bioactive secondary metabolites, potentially useful in developing new nematicidal and antimicrobial agents. This study focuses on extracting bioactive compounds from endosymbiotic bacteria associated with the marine sponge *Hyrtios erect sp.* (collected from NIOF Station, Hurghada, Red Sea, Egypt) using various organic solvents. *Bacillus* sp. was isolated and identified through 16 S rRNA gene sequencing. The biocidal activity of *Bacillus gotheilii* MSB1 extracts was screened against plant pathogenic bacteria, fungi, and nematodes. The n-butanol extract showed significant potential as a biological fungicide against *Alternaria alternata* and *Fusarium oxysporum*. Both n-hexane and ethyl acetate extracts exhibited negative impacts against the plant pathogenic bacteria *Erwinia carotovora* and *Ralstonia solanacearum*, whereas the n-butanol extract had a positive effect. Regarding nematicidal activity, ethyl acetate and n-butanol extracts demonstrated in-vitro activity against the root-knot nematode *Meloidogyne incognita*, which causes serious vegetable crop diseases, but the n-hexane extract showed no positive effects. The findings suggest that bioactive compounds from endosymbiotic bacteria associated with marine sponges, particularly *B. gotheilii* MSB1, hold significant potential as alternative biological control agents against plant pathogens. The n-butanol extract, in particular, displayed promising biocidal activities against various plant pathogenic fungi, bacteria, and nematodes. These results support further exploration and development of such bioactive compounds as sustainable, environmentally friendly alternatives to synthetic pesticides and fungicides in agricultural practices.

**Supplementary Information:**

The online version contains supplementary material available at 10.1186/s12896-024-00920-y.

## Introduction

Worldwide, approximately 40% of agricultural output is lost due to plant diseases, weeds, and pests. In the absence of pesticides, these crop losses would rise considerably. Furthermore, these crop-protection agents not only prevent pest damage but also play a key role in boosting yields [1].

Pesticides are toxic chemical compounds, combinations of substances, or biological agents that are deliberately introduced into the environment to manage, prevent, and eliminate populations of various plant-pathogenic organisms. These agents function by luring and then controlling pests. Over time, pests can evolve resistance to pesticides, generating different substances, including toxins that are detrimental to both plants and humans. The hazards linked to pesticide usage now outweigh their advantages. Moreover, pesticides have profound effects on non-target species, disrupting animal and plant biodiversity and impacting both aquatic and terrestrial food webs and ecosystems [2–4]. The unchecked application of pesticides has resulted in a decline of numerous terrestrial and aquatic animal and plant species [5]. This situation has underscored the significance of biologically active secondary metabolites in agricultural use, spurring the creation of new types of pesticides [6–9].

The marine environment has emerged as an exceptionally abundant source of powerful chemicals, with a multitude of species adapted to thrive in extreme conditions [10–14]. Marine organisms are particularly noteworthy for their capacity to produce bioactive compounds that exhibit significant antimicrobial, anticancer, anti-inflammatory, analgesic, immunomodulatory, antiallergy and antiviral properties [10, 15, 16].

Recent research underscores the significant potential of marine sponges as a source of bioactive compounds. Anteneh et al. [17]. discovered that marine sponges from South Australia host diverse bacteria capable of producing bioactive metabolites, with 70 out of 169 tested bacterial isolates showing antimicrobial activity against human pathogens such as Staphylococcus aureus and fungi. Notably, a novel compound from *Streptomyces* sp. was identified, highlighting these sponges’ potential as a source of new antibiotics. Similarly, Campana et al. [18]. revealed that sponges possess diverse microbial communities that play a critical role in the cycling of dissolved organic matter (DOM) in marine ecosystems. Using DNA-stable isotope probing and 16 S rRNA amplicon sequencing, they noted active DOM uptake by specific bacterial taxa, with PAUC34f, Poribacteria, and Chloroflexi identified as key players in organic matter degradation, while Nitrospirae may engage in mixotrophic metabolism.

Further, Sarjito et al. [19]. demonstrated the use of sponge-associated bacteria like *Bacillus* spp. to control vibriosis in shrimp, with chitosan encapsulation improving bacterial viability and shelf life. *Bacillus altitudinis* PH.1 encapsulated in chitosan showed high viability after 14 days at -20 °C and a strong anti-vibrio activity, suggesting chitosan as a promising preservation method. Wibowo et al. [20]. highlighted marine bacteria as a rich source of novel bioactive compounds against drug-resistant pathogens. Research between January 2016 and December 2021 focused on secondary metabolites, with *Streptomyces* and other Actinobacteria revealing compounds effective against MRSA, VRE, MDR-TB, and amphotericin B-resistant *Candida albicans*, indicating their potential in new drug development.

Additionally, Bibi et al. [21] noted sponges as rich sources of bioactive natural products synthesized by symbiotic bacteria, with approximately 5,300 natural compounds identified to date. These products are often produced due to environmental competition for space and nutrients. The review emphasizes sponge-microbe interactions, highlighting the significant industrial and pharmaceutical potential of sponge-associated bacteria. Hentschel et al. [22]. highlighted the diverse microbial communities within marine sponges, comprising close to 30 bacterial phyla and several archaeal lineages, which can constitute up to 35% of sponge biomass. The sequencing of the *Amphimedon queenslandica* genome has provided insights into animal evolution and sponge-symbiont interactions, reinforcing the importance of marine sponges in studying host-microbe relationships.

Sponges are known to produce a wide array of chemically diverse bioactive substances, such as sterols, terpenes, nucleosides, cyclic peptides, and alkaloids [10, 23]. Although sponges themselves synthesize many of these valuable compounds, their microbial symbionts also play a crucial role in their production [24]. A significant part of the biomass of many marine sponges is composed of dense and genetically diverse microbial communities [24, 25], which include bacteria, archaea, fungi, and microalgae [26–28].

Studies have shown that *Bacillus* species dominate the microbial communities within marine sponges [29–32]. These bacteria are particularly noted for producing a variety of antimicrobial peptides with diverse chemical structures, making them central to bacteriocin research. *Bacillus* strains exhibit the broadest array of agriculturally beneficial compounds with significant potential [33, 34]. They are also renowned for producing various antifungal compounds that control or suppress fungal pathogens [35–37]. Recently, volatile organic compounds (VOCs) synthesized by *Bacillus* species have been explored as a novel approach to managing plant fungal diseases [38–44]. These VOCs can diffuse between soil particles and spread extensively from their application sites, exerting inhibitory effects without direct contact between the VOC-producing microorganisms and target pathogens [45, 46]. Their potent antifungal properties, coupled with their safety for the environment and human health, make VOCs a promising and sustainable alternative to traditional fungicides for future plant pathogen control [47–49].

*Alternaria alternata* is a fungal pathogen responsible for early blight disease in tomatoes, potatoes, tobacco, and various other vegetables and crops, resulting in substantial agricultural losses [50, 51]. For instance, potatoes, one of the most crucial crops globally, can experience up to 80% annual yield reductions due to early blight in specific regions [38, 39]. At present, chemical fungicides are the most effective means to manage early blight. However, excessive and improper use of these fungicides has led to the development of resistant pathogens, threatening both food safety and human health [52, 53]. *Fusarium* species are another group of phytopathogens that impact numerous economically significant crops. Among them, *Fusarium oxysporum* is particularly widespread, with over 120 reported *formae speciales* [54]. To combat these diseases, a variety of agronomic practices have been devised, including biological control, cultural practices, and chemical interventions.

In this study, we isolated, purified, and characterized several active ingredients produced by marine bacteria, aiming to explore their potential as alternatives to chemical pesticides. The effectiveness of these isolated compounds was evaluated against two pathogenic fungi; *Alternaria alternata* and *Fusarium oxysporum*, as well as the root-knot nematode *Meloidogyne incognita*.

## Materials and methods

All chemicals and microbial media used in this study were sourced from Becton Dickinson **(Sparks**, **MD**, **USA)** and Loba Chemie PVT. LTD **(Mumbai**, **India)**, with additional chemicals purchased from Sigma-Aldrich **(St. Louis**, **MO**, **USA)**.

### Sponge collection, identification and storage

A marine sponge sample was collected from the Red Sea near Hurghada, Egypt, at a depth of 2 m, close to the National Institute of Oceanography and Fisheries Station (coordinates: N 27 17 07.45, E 33 46 26.50). The sponge was identified as *Hyrtios erecta* (Order Dictyoceratida, Family Thorectidae) thanks to Prof. Rob. W. M. van Soest from the Department of Marine Zoology at the Netherlands Centre for Biodiversity [55, 56]. To cleanse the specimens of loosely attached bacteria, they were initially rinsed with sterilized artificial seawater (ASW) [57] and then underwent surface sterilization using 70% alcohol. Following sterilization, the sponge samples were rapidly placed on dry ice, frozen, and subsequently stored at -20 °C.

### Preparation of the bacterial culture media

Marine Nutrient Agar (MNA) was employed as a general nutrient-rich medium to culture various heterotrophic marine bacteria. These bacteria were subsequently subcultured on ISP Agar medium following established protocols [58, 59]. Each medium was supplemented with NaCl at a concentration of 2% (w/v). To inhibit fungal growth, nystatin was added at a concentration of 25 µg/ml, as described by Webster et al. [60]. The plates were then filled, inverted, and stored at 4 °C until further use.

### Isolation and purification of the suspected bacterial strain(s)

A fresh sponge sample, approximately 1 cm³ in size, was thoroughly milled in a sterile mortar with 10 ml of sterile artificial seawater (ASW) for two to three minutes. The resultant homogenate was transferred into a sterile 15 ml test tube, creating a 10 ^− 1^ dilution. A 100 µL aliquot from each dilution series was spread onto various isolation media using a 10-fold dilution approach. The plates were then aerobically incubated for 1–2 weeks at 30 °C. Unique colony morphotypes were repeatedly selected and sub-cultured to achieve pure cultures, identified by their colony homogeneity.

The purified isolates were subsequently inoculated into ISP Medium 2 (ISP2). The liquid cultures were incubated at 37 °C in a shaking incubator set at 160 rpm for 48–72 h. Following the incubation period, cells were harvested by centrifugation at 6000 × g for 15 min.

### Morphological and molecular identification of selected bacterial strain using 16 S rRNA gene

The *Bacillus* sp. isolate underwent further identification through 16S rRNA gene sequence analysis, following a series of morphological, microscopic, and biochemical characterizations. The polymerase chain reaction (PCR) amplification of the 16S rRNA gene was performed using specific primers: 27f (5’-AGAGTTTGATCCTGGCTCAG-3’) and 1492r (5’-GGTTACCTTGTTACGACTT-3’) [61]. The entire genome was amplified using a PCR system cycler from Creacon **(Holland**, **Inc.)**.

### Extraction of bioactive secondary metabolites

Under optimal conditions, *B. gottheilii* MSB1 was cultured in liquid ISP2 media at 28 °C with continuous shaking at 200 rpm for 48 h. The cultures were then centrifuged at 6000 rpm for 15 min at 4 °C to separate the cells from the supernatant.

Subsequently, 1 L of the culture filtrate was subjected to liquid-liquid extraction using 250 ml each of ethyl acetate and n-butanol. The extractions were performed sequentially, starting with ethyl acetate, followed by n-butanol.

Each organic phase was individually separated and dried over anhydrous sodium sulphate. The drying process was carried out at room temperature (approximately 25 °C) to prevent any degradation of the bioactive compounds. The solvents were concentrated using a rotary evaporator (**ROTAVAP**, **Büchi**, **Switzerland**) conducted under reduced pressure at 40 °C. Each evaporation step took approximately 1 h to ensure complete removal of solvents.

The concentrated residues were then reconstituted in 10 ml of methanol, achieving a concentration of 1.7 mg/ml for the ethyl acetate extract and 1.5 mg/ml for the n-butanol extract. This resulted in a yield of crude precipitates of 17 mg from the ethyl acetate extraction and 15 mg from the n-butanol extraction.

### GC/MS Analysis of *B. gottheilii* MSB1 Extracts

The GC/MS apparatus was utilized to identify biologically active compounds from the *B. gottheilii* MSB1 isolate. This analysis was conducted using a Gas Chromatograph **(Agilent 7690 A)** and a Mass Spectrometer (**Agilent 5975 C with Triple Axis Detector)** at the National Institute of Oceanography and Fisheries’ Alexandria Branch in Egypt. Specific conditions included: the mass spectrometer detector operating at 70 eV with a source temperature of 325 °C. The injector temperature was maintained at 300 °C, utilizing a splitless injection mode with an injection volume of 1 µl and a purge time of 2 min. Helium served as the carrier gas at a rate of 1.22 ml/min [62]. Separation was conducted on an HP-5MS 5% Phenyl Methyl Siloxane Column **(Agilent 19091 S-433)** measuring 30 m x 250 μm x 0.25 μm. The oven temperature program began with holding at 90 °C for 1 min, followed by an increase of 8 °C/min to reach 205 °C, held for another minute. It was then increased at 5 °C/min to 240 °C for one minute, and finally at 8 °C/min attaining a peak temperature of 300 °C, which was maintained for 30 min. The total duration of the run was 61.875 min. Following the run, the resulting mass spectra of the components were analyzed and compared.

### Fungal isolation, purification and identification

Fungi were extracted from plant leaves and subsequently cultivated in a potato dextrose agar (PDA) medium, which was composed of 200 g of potato, 20 g of glucose, and distilled water for up to 1 L. The cultures were then incubated at 28 °C for a duration of 7 days. Total RNA was isolated from both treated and untreated fungal samples, with the treatment involving an n-butanol extract, following the protocol established by Chomczynski et al. [63].

### Reverse transcription reaction-polymerase chain reaction (RT-PCR)

Complementary DNA (cDNA) was synthesized by reverse transcribing total RNA in a 20 µL reaction mixture. The components included: 3 µL of total RNA, 5 µL of oligo(dT) primer (10 pmol/µL), 2.5 µL of dNTPs (10 mM), 2.5 µL of buffer (10x), 0.3 µL of Reverse Transcriptase, and 6.7 µL of sterile distilled water to achieve a final volume of 20 µL. The mixture was gently mixed and placed in a thermocycler. The amplification program consisted of incubation at 37 °C for 1 h, followed by enzyme inactivation at 65 °C for 10 min, and then cooling to 4 °C. The resulting cDNA was stored at -20 °C for further use.

In this study, four primers (*PR2*, *PR3*, *PR4*, and *PR5*) were employed, as detailed in Table 1. The Real-Time PCR reaction mixture included: 10 µL of SYBR Green, 1 µL of 10 pmol/µL forward primer, 1 µL of 10 pmol/µL reverse primer, 1 µL of cDNA (50 ng), and sterile distilled water to make up a total volume of 20 µL. The Real-Time PCR cycling conditions were: an initial denaturation at 95 °C for 10 min; followed by 45 cycles at 95 °C for 10 s, annealing at 60 °C for 20 s, and extension at 72 °C for 20 s. Data acquisition occurred during the extension phase. This reaction was analyzed using the Rotor-Gene 6000 system from Qiagen (USA).

The difference in quantification cycle values (ΔΔCT) between the reference and the target (treated) was calculated. The threshold cycle for each gene was determined using automated threshold analysis on the ABI system. Gene expression levels were quantified according to the method described by Livak et al. [64]. The C_T_ value for each target gene was normalized to the C_T_ value of the reference gene to obtain ΔC_T(target)_.

**Table 1:** The specific primers of defense genes used in real-time PCR

Genes	Primers	Sequence 5`-----3`	Reference
*β-actin*	F: R:	5`-ATGCCATTCTCCGTCTTGACTTG-3` 5`-GAACCTAAGCCACGATACCA-3`	[65]
*PR2*	F: R:	5`-TCACCAAACTATTGGATTTCAA-3` 5`-GACTCAATTTTTGACTTCTTAATCC-3`	[66]
*PR3*	F: R:	5`-ACTGGAGGATGGGCTTCAGCA-3` 5`-TGGATGGGGCCTCGTCCGAA-3`	[67]
*PR4*	F: R:	5`-GACAACAATGCGGTCGTCAAGG-3` 5`-AGCATGTTTCTGGAATCAGGCTG-3`	[68]
*PR5*	F: R:	5`-ATGGGGTAAACCACCAAACA-3` 5`-GTTAGTTGGGCCGAAAGACA-3`	[66]

### Statistical analysis

In this study examining the antimicrobial and nematicidal activities of *B. gottheilii* MSB1 extracts, we utilized statistical approaches to substantiate our findings and ensure scientific rigor. Specifically, the inhibition zones and nematicidal mortality percentages were subjected to one-way analysis of variance (ANOVA) to detect significant differences among treatments. This approach enabled us to assess the relative effectiveness of each extract type (n-butanol, n-hexane, and ethyl acetate) against plant pathogens and nematodes. When ANOVA indicated significant differences at a confidence level of *p* < 0.05, Tukey’s Honest Significant Difference (HSD) test was subsequently applied. This post-hoc analysis allowed us to determine pairwise differences among group means, providing a clearer understanding of the superior efficacy of particular extracts over others.

Additionally, statistical metrics such as least significant difference (LSD) and confidence intervals were calculated to enhance the reliability of our findings, offering insights into the precision and consistency of the observed effects. Statistical analyses were conducted using SPSS software (version 25), facilitating a robust examination of the dataset and ensuring data integrity. The use of these statistical tools enabled us to critically evaluate the potential of *B. gottheilii* MSB1 extracts as viable alternatives to conventional chemical pesticides, grounding our claims in quantitative evidence and supporting the exploration of environmentally friendly biocontrol solutions.

## Results

The purpose of this study was to assess the ability of marine bacteria associated with sponges to produce antifungal and nematocidal compounds.

### Phylogenetic analysis of the selected *bacillus sp.* isolate

**Fig. 1:**
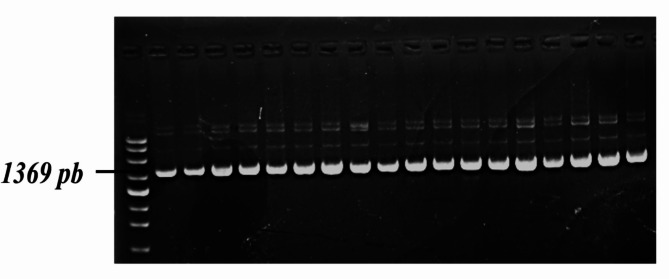
Agarose gel electrophoresis of the amplified region of the isolate *Bacillus sp*. specific genes with approx. 1369 bp. The full, uncropped gel is shown in the supplementary file, Fig. S1

**Fig. 2:**
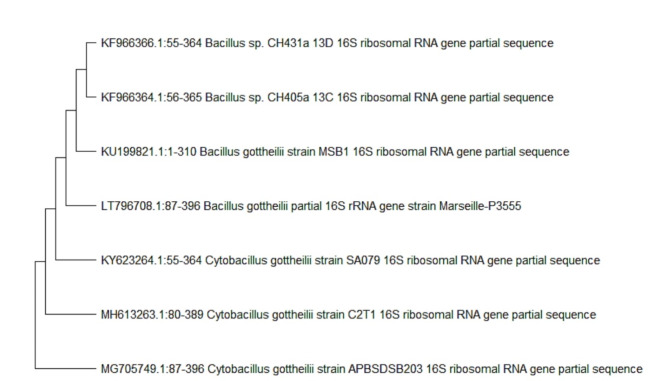
phylogenetic tree for *B. gottheilii* MSB1 according to specific gene sequencing data

**Fig. 3:**
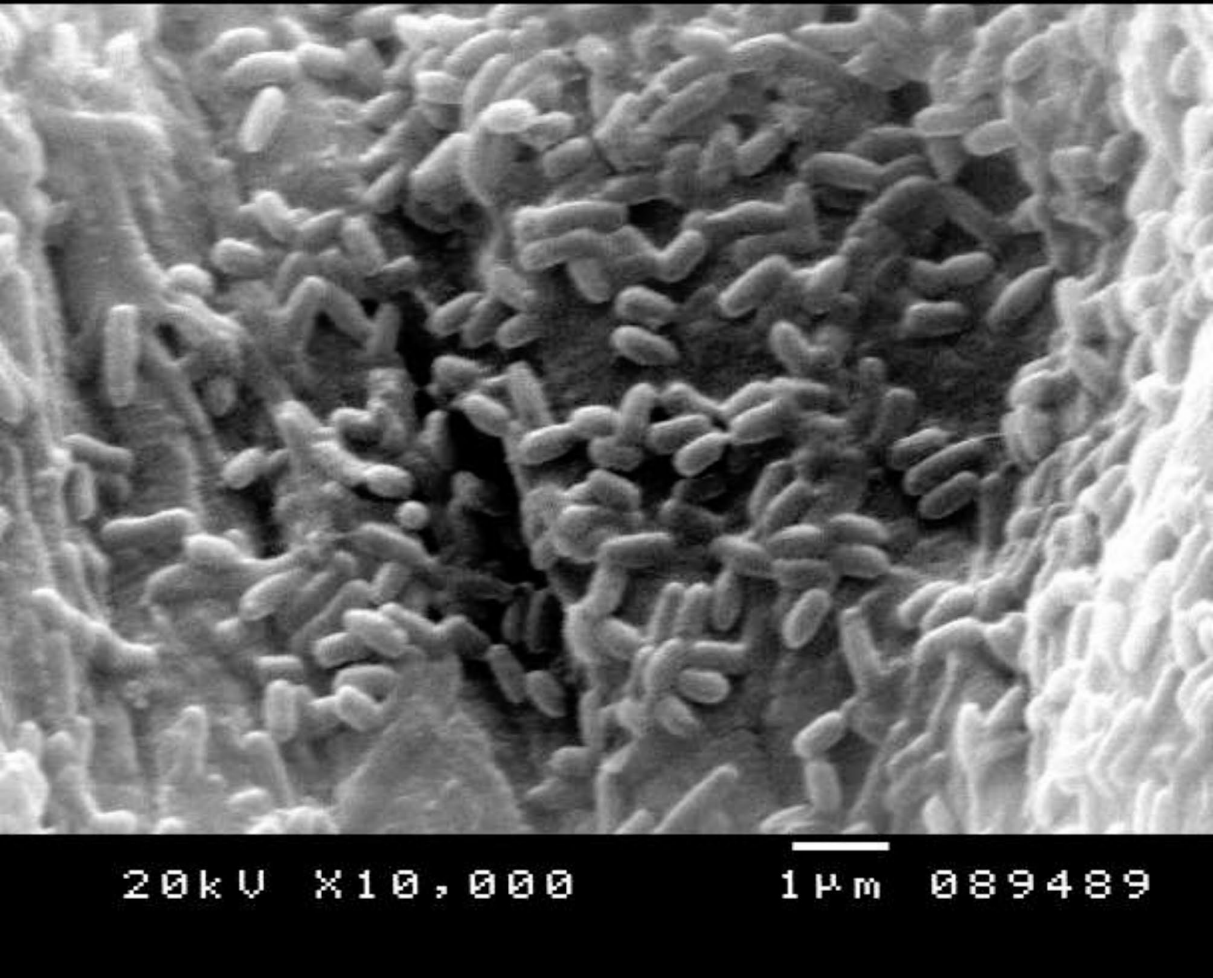
*B. gottheilii* MSB1 cells by scanning electron microscope (SEM)

The 16 S rRNA gene sequences were used to identify the chosen isolate. Nucleotide sequence analysis was conducted using the BlastN tool on the NCBI server. The *Bacillus* sp. isolate, identified as *B. gottheilii* MSB1, has a sequence length of 1369 bp (Figs: 1, 2 and 3). The sequence of the identified bacterial strain has been submitted to GenBank with the accession number KU199821.

### GC/MS analysis of *B. gottheilii* MSB1 ethyl acetate extract

Using the GC/MS technique, four different chemicals were identified in the ethyl acetate extract of *B. gottheilii* MSB1 (Table 2; Fig. 3). The identified compounds include 3,5-Di-tert-butylphenol (20.4%), Ethyl 14-methyl-hexadecanoate (4.99%), Phthalic acid isobutyl octadecyl ester (4.83%), and Ethyl 12-oxododecanoate (4.65%).

Figure 3. *B. gottheilii* MSB1 cells by scanning electron microscope (SEM).

**Table 2:** GC/MS analysis of ethyl acetate extract of BGMSB1

Peak #	Compound	*R*_t_ (min)	Molecular Formula	Molecular Weight (g/ mol)
1	2,4-Di-tert-butylphenol	13.88	C_14_H_22_O	206.32
2	Ethyl 14-methyl hexadecanoate	19.67	C_19_H_38_O_2_	298.50
3	Phthalic acid, isobutyl octadecyl ester	20.08	C_30_H_50_O_4_	474.72
4	Ethyl 12-oxododecanoate	22.14	C_14_H_26_O_3_	242.35

### GC/MS analysis of *B. gottheilii* MSB1 n-butanol extract

The chemical composition of the n-butanol extract from *B. gottheilii* MSB1 is detailed in Table 3. Using GC/MS, seventeen components were identified in the n-butanol extract. The predominant compounds were Benzeneacetamide (70.0%), 2-Hydroxy-1-[(palmitoyloxy)methyl]ethyl palmitate (36.4%), 3-Methylbutanamide (23.1%), 2,6-Di-tert-butylphenol (9.23%), Ethyl cis, cis-9,12-octadecadienoate (11.7%), (7Z)-7-Nonenamide (9.27%), 2-Methylpropyl hexadecanoate (6.68%), 2-Propyl-1-heptanol (6.03%), 1-Hexadecanol (5.08%), 3,3-Dimethyl-6-(methylsulfanyl)-8-(4-morpholinyl)-3,4-dihydro-1 H-thiopyrano[3,4-c] pyridine-5-carbonitrile (4.65%), (5E)-5-Icosene (3.51%), and (9E)-9-Hexadecen-1-ol (3.19%).

**Table 3:** GC/MS analysis of *B. Gottheilii* MSB1 n-butanol extract

Bacillus	Compound	*R*_t_ (min)	Molecular Formula	Molecular Weight (g/ mol)
1	3-Methylbutanamide	5.64	C_5_H_11_NO	101.15
2	2-Propyl-1-heptanol	6.10	C_10_H_22_O	158.28
3	4-Fluorophenyl butyrate	6.60	C_10_H_11_FO_2_	182.19
4	(7Z)-7-Nonenamide	7.33	C_9_H_17_NO	155.24
5	cis-3-Dodecene	8.33	C_12_H_24_	168.32
6	1-Hexadecanol	11.78	C_16_H_34_O	242.44
7	Benzeneacetamide	12.42	C_8_H_9_NO	135.16
8	2,6-Di-tert-butylphenol	13.94	C_14_H_22_O	206.32
9	(9E)-9-Hexadecen-1-ol	15.13	C_16_H_32_O	240.42
10	(5E)-5-Icosene	18.52	C_20_H_40_	280.53
11	4-Amino-6-hydroxy-2-methyl-5-nitrosopyrimidine	21.50	C_5_H_6_N_4_O_2_	154.13
12	Butyl 13-methyltetradecanoate	22.12	C_19_H_38_O_2_	298.50
13	2-Hydroxy-1-[(palmitoyloxy)methyl] ethyl palmitate	25.05	C_35_H_68_O_5_	568.91
14	2-methylpropyl hexadecanoate	25.72	C_20_H_40_O_2_	312.53
15	3,7,11-Trimethyl-1-dodecanol	25.84	C_15_H_32_O	228.41
16	3,3-Dimethyl-6-(methylsulfanyl)-8-(4-morpholinyl)-3,4-dihydro-1 H-thiopyrano[3,4-c]pyridine-5-carbonitrile	26.01	C_16_H_21_N_3_OS_2_	335.49
17	Ethyl cis, cis-9,12-octadecadienoate	28.63	C_20_H_36_O_2_	308.50

While this analysis successfully identifies several compounds within the extract, it remains essential to delve deeper into how these compounds exert their biological effects. Future research should prioritize elucidating the underlying mechanisms by which these compounds may disrupt fungal or bacterial growth. Understanding these mechanisms will significantly enhance the study’s scientific contribution and provide insight into potential applications for these bioactive compounds in medical or agricultural fields.

### Antibacterial and nematicidal activity of *B. gottheilii* MSB1 extracts

The antibacterial activity of n-hexane and ethyl acetate extracts from *B. gottheilii* MSB1 showed no significant impact against plant pathogenic bacteria such as *Erwinia carotovora* and *Ralstonia solanacearum*. However, the n-butanol extract demonstrated positive effects, inhibiting these bacteria with inhibition zones of 17 mm and 19 mm, respectively. *Erwinia carotovora* causes disease in a wide variety of agricultural and horticultural crops, including carrots, cabbage, cucumbers, onions, tomatoes, lettuce, and ornamental plants such as iris. *Ralstonia solanacearum* is responsible for causing brown (wilt) potato rot. These pathogens continuously attack potatoes and other vegetable crops both in fields and in storage, posing significant agricultural concerns (Table 4).

**Table 4:** The antibacterial activity for plant pathogenic bacteria

BGMSB1 extracts	Diameter of inhibition zone (mm)	
	Plant pathogenic isolate	
	Erwinia Carotovora	Ralstonia solanacearum
n-Hexane	-	-
Ethyl acetate	-	-
n-Butanol	17	19

In addition, ethyl acetate and n-butanol extracts exhibited in-vitro nematicidal activity against the root-knot nematode (*Meloidogyne incognita*), which causes serious diseases in vegetable crops. The ethyl acetate extract showed a 90.5% reduction in nematode activity after 12 h and 100% reduction after 24 h and 7 days. The n-butanol extract showed an 85.7% reduction after 12 h and 24 h, and a 90.5% reduction after 7 days. In contrast, the n-hexane extract showed no significant effects (Table 5). We employed a negative control consisting of only the testing medium without any extracts to establish a baseline for natural nematode mortality, which remained consistently low (4.2% mortality across all time points), confirming that the observed reductions were due to the active compounds in the extracts.

**Table 5:** In-vitro nematicidal activity (*Meloidogyne incognita*), data are means of 3 replicates

No.	Treatment	Con.	(J_2_ mortality %)					
			12 h		24 h		7 days	
			L	*R* (%)	L	*R* (%)	L	*R* (%)
1	Negative control (MI)	-	4.2^a^	-	4.2^a^	-	4.2^a^	-
2	MI + Nemaphose 40% (Positive_control)	-	0.6^b^	85.7	0.2^bc^	95.2	0.00^b^	100
3	MI + H extract	-	0.4^b^	-	0.4^b^	-	0.4^b^	-
4	MI + E extract	S	0.4^b^	90.5	0.0^c^	100	0.0^b^	100
5	MI + B extract	S	0.6^b^	85.7	0.6^b^	85.7	0.4^b^	90.5

**S**: standard. ^**a, b,c**^Means with the same letters (s), in each column, are not significantly different at (*p* ≤ 0.05). **Con.**: Concentration; **J2**: Second-stage juvenile; **L**: Lethality (percentage of J2 mortality); R (%): Reduction percentage; **MI**: Meloidogyne incognita; **H extract**: Herbal extract; E extract: Extract of compound ‘E’; **B extract**: Extract of compound ‘B’

### Antifungal activity of *B. gottheilii* MSB1 n-butanol extract

The in vitro antifungal activity of the n-butanol extract against the plant pathogenic fungi *Fusarium oxysporum* and *Alternaria alternata* is presented in Table 6, with results expressed as IC50 values. Most of the tested compounds showed inhibitory effects against the fungi. Concerning *Fusarium oxysporum*, the extract exhibited significantly potent antifungal activity with an IC50 of 0.086 mg/L. Similarly, for *Alternaria alternata*, the extract demonstrated an IC50 of 0.061 mg/L (Figs; 4 and 5).

**Table 6:** The in vitro antifungal activity of *B. Gottheilii* MSB1 n-butanol extract against *F. Oxysporum* AND *A. Alternaria* by Mycelia radial growth technique

Formulations	EC_50_^1^ (mg/L)	95% confidence limits		Slope^2^ ± SE	Intercept^3^ ± SE	(χ^2^)^4^
		Lower	Upper			
Alternaria	0.830	0.113	1.759	1.48 ± 0.353	0.120 ± 0.329	1.77
Fusarium	1.761	0.818	2.625	2.021 ± 0.348	-0.497 ± 0.31	0.931

^**1**^The concentration causing 50% mycelia growth inhibition. ^**2**^Slope of the concentration-inhibition regression line ± standard error.^**3**^Intercept of the regression line ± standard error.^**4**^Chi square value

**Fig. 4:**
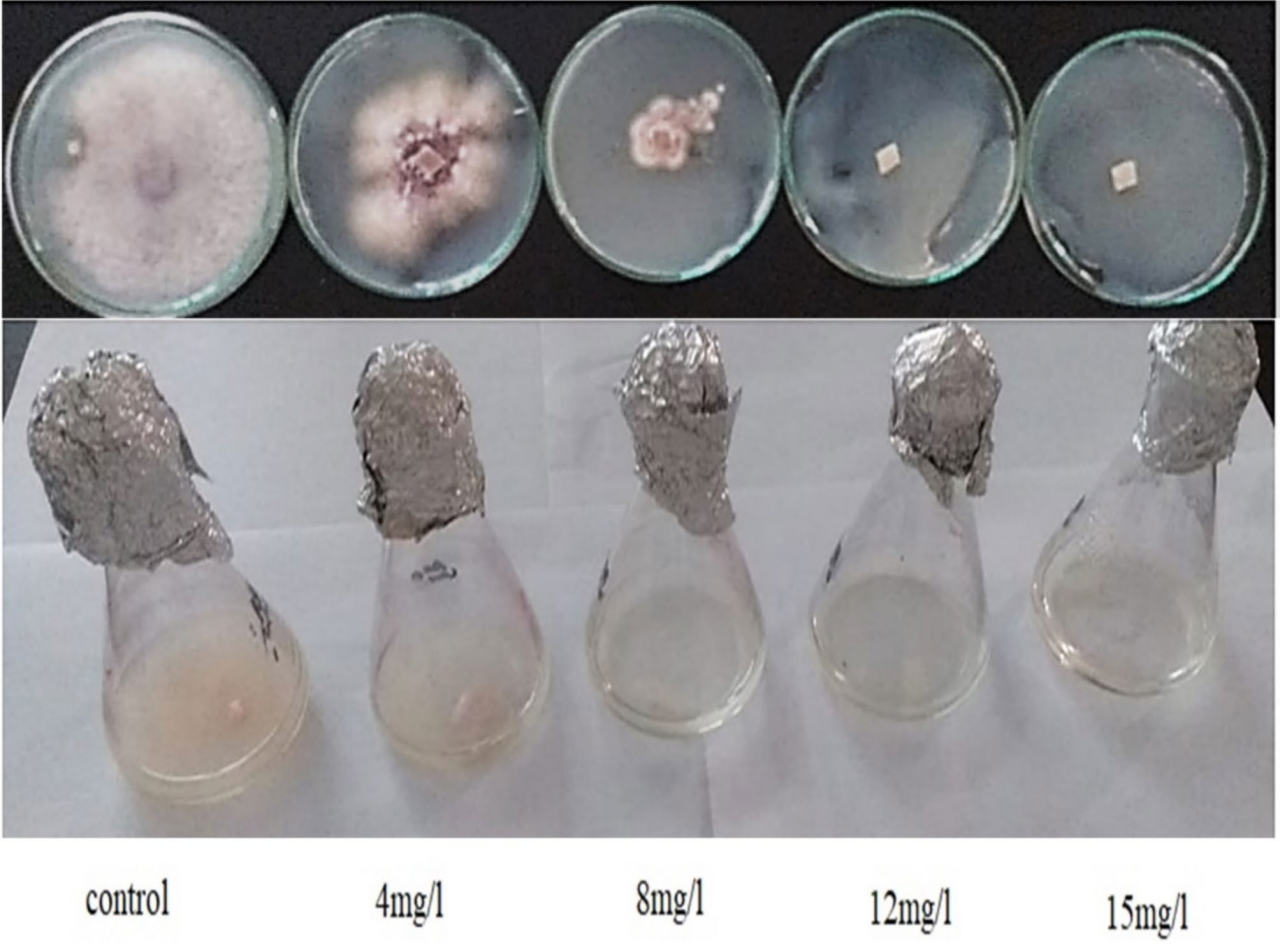
The antifungal activity of the *B. gottheilii* MSB1 n-butanol extract (from left to right, 0, 4, 8, 12and 15 mg/L respectively) against *F. oxysporum*

**Fig. 5:**
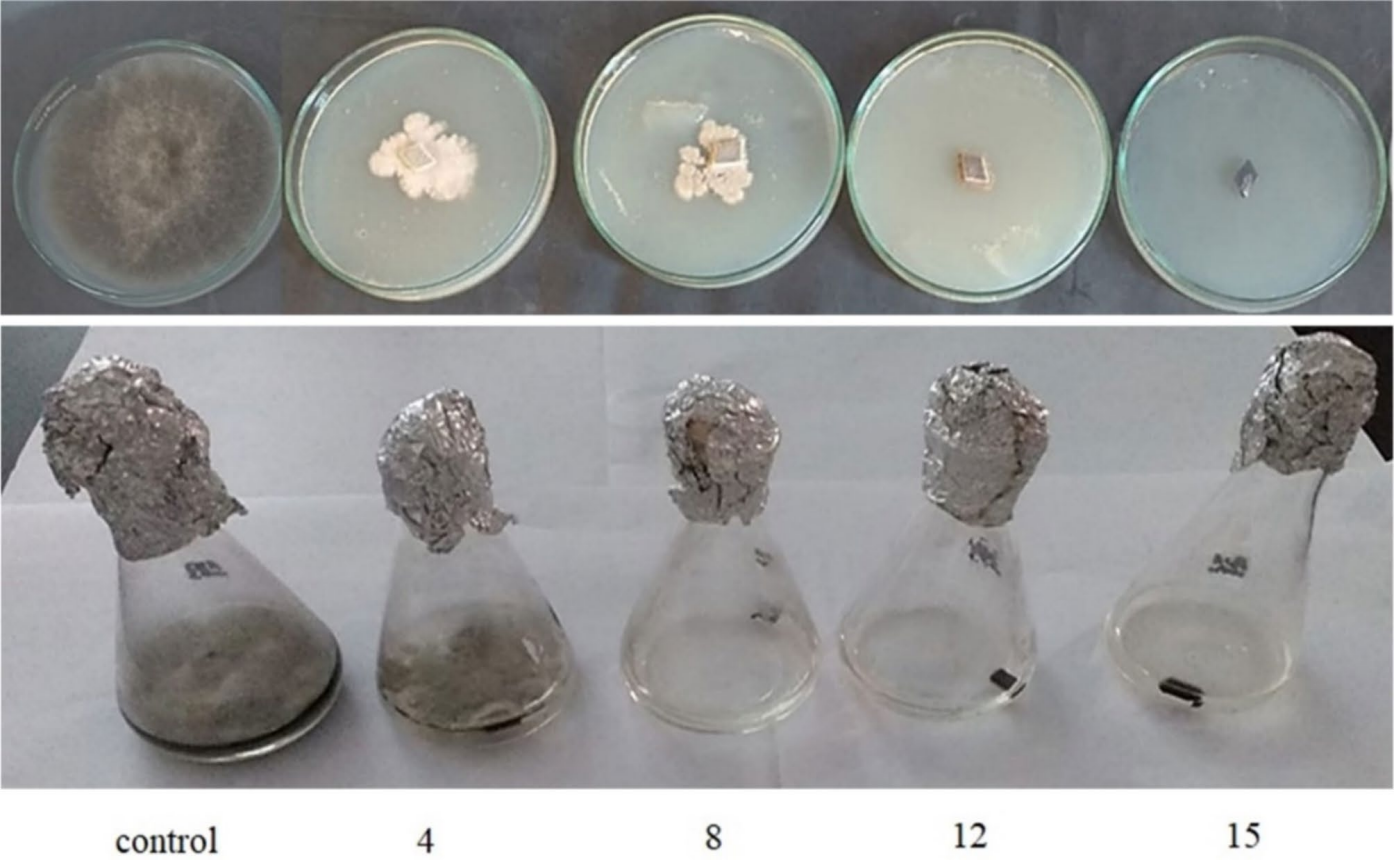
The antifungal activity of the *B. gottheilii* MSB1 n-butanol extract (from left to right, 0, 4, 8, 12 and 15 mg/L respectively) against *A. Alternaria*

### Quantitative real-time PCR (QRT-PCR)

**Fig. 6:**
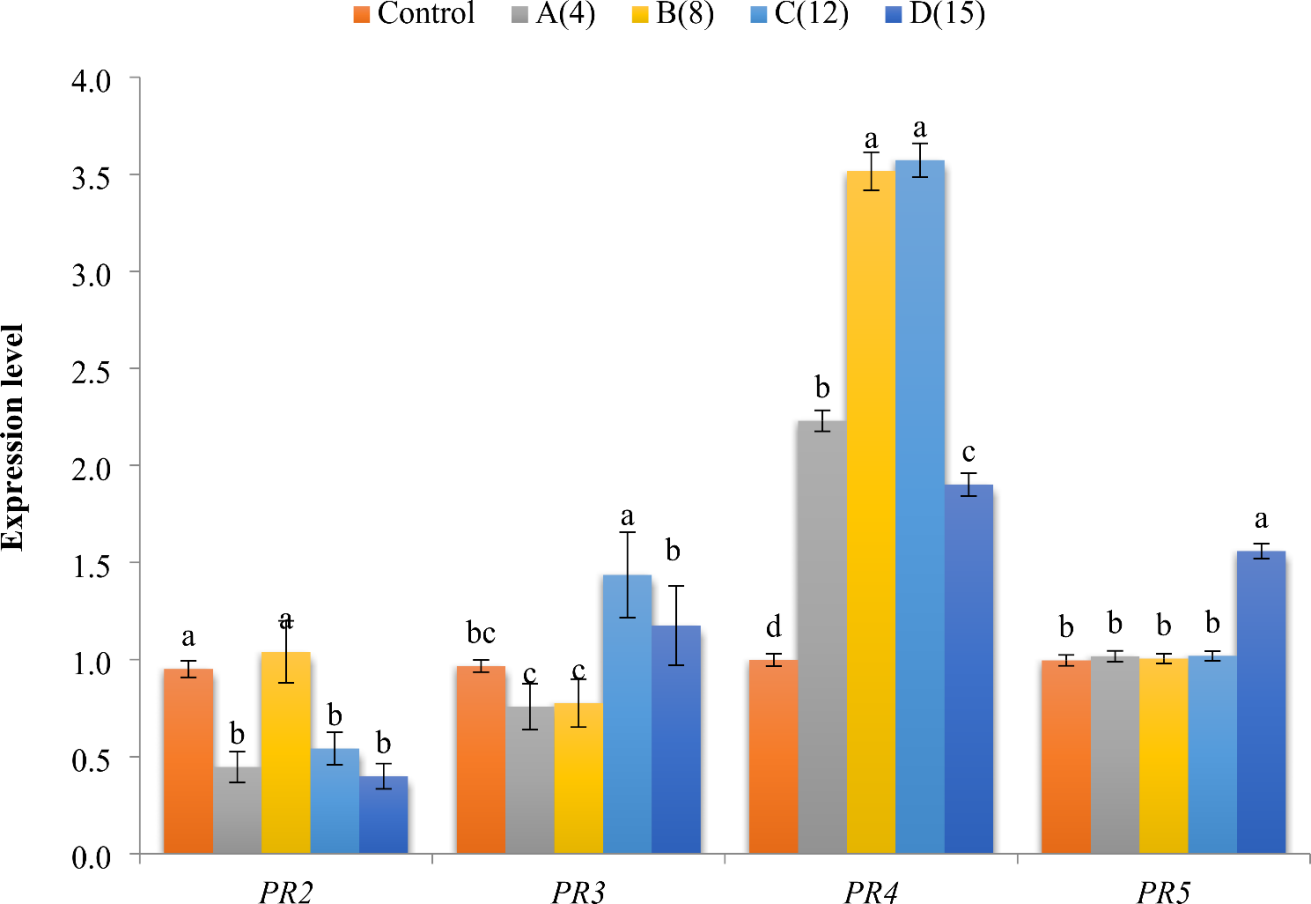
Histogram of the quantitative estimation for *PR2*, *PR3*, *PR4* and *PR5* gene expressions in *Alternaria* treated with *B. gottheilii* MSB1 n-butanol extract

**Fig. 7:**
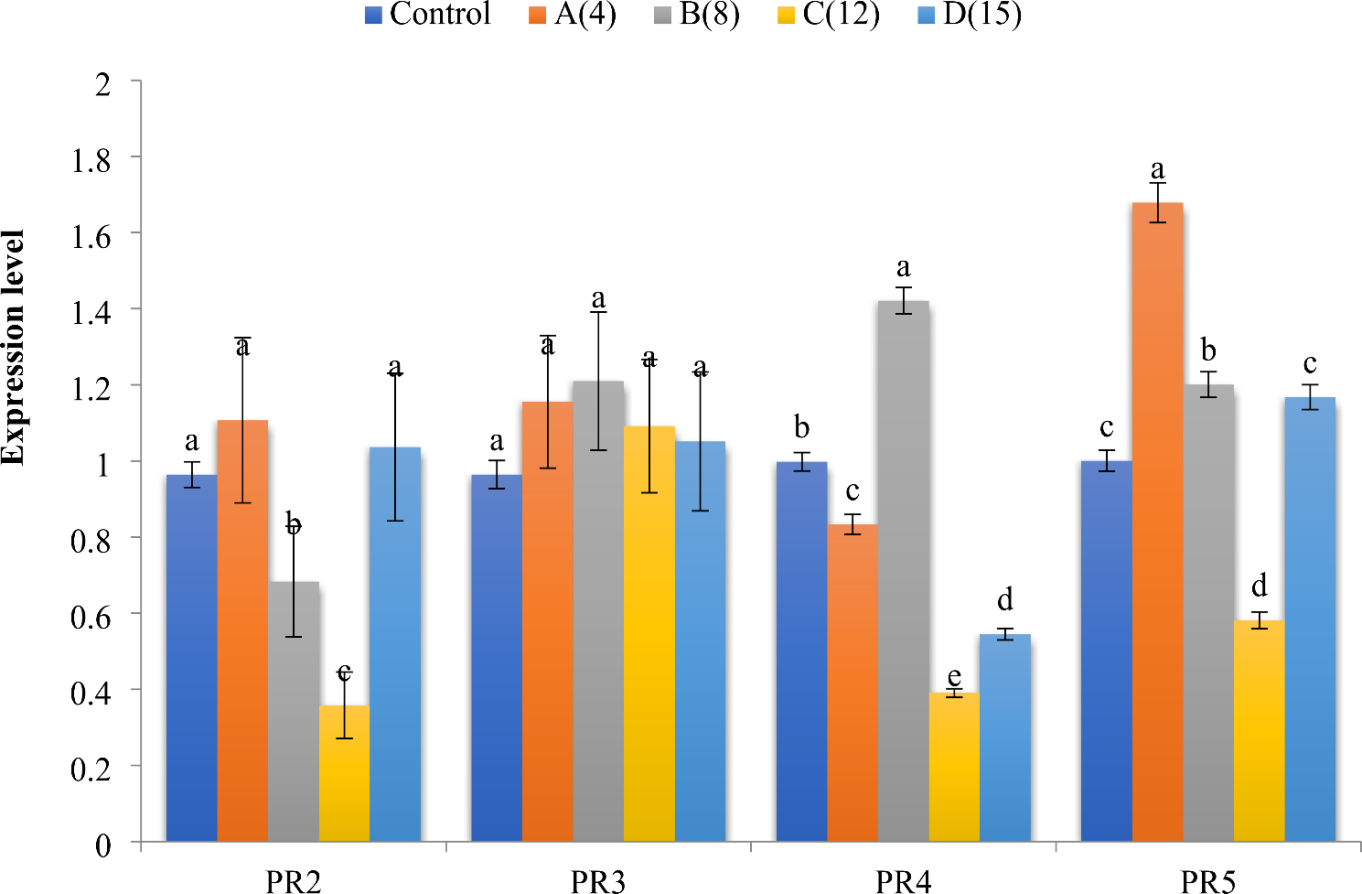
Histogram of the quantitative estimation for *PR2*, *PR3*, *PR4* and *PR5* gene expressions in *Alternaria* treated with *B. gottheilii* MSB1 n-butanol extract

Real-time PCR was employed to quantify the relative mRNA levels of four related protein genes in two plant pathogenic fungi, *Alternaria alternata* and *Fusarium oxysporium*, treated with extract No. 3. The analyzed defense genes include β-1, 3-glucanases (*PR2*), *PR3*, *PR4*, and thaumatin-like proteins (*PR5*). Results were normalized using the elongation factor β-actin gene as a reference or housekeeping gene. The quantitative analysis revealed that *PR4* expression had the highest level with *A. alternata* (3.58) at times 8 and 12, respectively. Conversely, the lowest values (12) were observed with *A. alternata* for *PR2*. Moreover, for F. oxysporium, the highest expression levels were noted for *PR5*, *PR3* and *PR2* when treated with concentrate 4, whereas the lowest expression (*PR4*) was detected when treated with concentrate 12 (Figs: 6 and 7).

## Discussion

Plant diseases caused by various microorganisms, including viruses, bacteria, fungi, protozoa, and nematodes, significantly affect agricultural practices, resulting in substantial crop losses [69, 70]. Fungal pathogens are a primary cause of plant diseases, infecting a majority of plants. Agrochemicals are vital in plant disease management to achieve a sustainable and productive agricultural system [71, 72]. However, the extensive use of chemicals has detrimental effects on human health, ecosystem functionality, and agricultural sustainability. Sustainable agriculture can be achieved by reducing or eliminating the use of fertilizers and agrochemicals, thereby minimizing environmental impact [73, 74].

Despite considerable efforts in managing plant pathogens, there is still significant potential to discover stable solutions. Various agronomical, biochemical, chemical, cultural, and biotechnological approaches have been employed to address plant pathogen issues, each with varying degrees of success. The use of bioactive secondary metabolites produced by biocontrol agents has emerged as a promising, eco-friendly approach for managing plant pathogens [75].

Sponge-associated bacteria and fungi are known to produce antimicrobial compounds with unique biologically important properties [76]. The majority of these antimicrobial compounds are produced by bacteria (90%), with fungi accounting for approximately 10% [77]. The interactions between sponges and bacteria in the marine environment are not well understood, but symbiotic relationships are generally believed to exist between sponges and microorganisms [78, 79].

Symbiotic functions attributed to microbial flora include nutrient acquisition, stabilization of the sponge skeleton, processing of metabolic waste, and secondary metabolite production [80]. Some bacteria are also thought to chemically defend their host against microbial infection [81]. Our study has confirmed that *Bacillus sp*. is predominantly represented, and its occurrence in the marine environment is well documented [82].

Sponge-associated *Bacillus* species, such as *Bacillus cereus*, *Bacillus flexus*, *Bacillus pumilus*, *Bacillus licheniformis*, *Bacillus megaterium*, *Bacillus amyloliquefaciens*, and *Bacillus subtilis*, offer significant potential as sources of antimicrobial substances [37, 83–92]. Numerous microbial isolates from these species have been reported to inhibit pathogenic reference strains in vitro and synthesize active substances effective against various infectious agents [39, 93–95]. These Bacillus species produce a wide array of pharmacologically and agriculturally active compounds and are recognized as industrially important microorganisms for their ability to generate numerous novel secondary metabolites [96, 97].

Optimizing culture conditions is essential to achieve high yields of these metabolites. Therefore, an attempt was made to optimize nutritional sources, including carbon, nitrogen, and minerals, as well as environmental factors such as time, pH, and temperature, for the production of antimicrobial metabolites by Bacillus strains [98, 99]. Competition among microbes for space and nutrients in the marine environment serves as a powerful selection pressure, encouraging marine microorganisms to produce natural products with significant agricultural, medical, and industrial applications [100].

The marine environment remains a critical area of research for scientists globally. The extreme conditions of temperature and salinity in marine ecosystems provide a unique and largely untapped microbiome [101]. These unique environmental conditions offer opportunities for the exploration of new microbes and their secondary metabolites [102].

The marine sponge *Hyrtios erecta* was found to harbor potent bacterial strains, particularly *B. gottheilii* MSB1, when screened against various pathogenic bacteria and fungi. The presence of antimicrobial activity was confirmed [103, 104]. Symbiotic and endophytic microorganisms have been identified as promising natural sources of antimicrobial and biocontrol agents (herbicides, pesticides), offering a viable approach to reducing the use of agrochemicals [105, 106].

GC/MS analysis of ethyl acetate and n-butanol extracts of *B. gottheilii* MSB1 revealed a diverse array of bioactive secondary metabolites with antimicrobial properties, which likely contribute to its strong antagonistic activity against *Fusarium oxysporum* and *Alternaria alternata* [107, 108]. Additionally, these extracts exhibited significant in-vitro nematicidal activity against the root-knot nematode (*Meloidogyne incognita*), with reduction rates of 90.5% after 12 h and 100% after 24 h and 7 days for ethyl acetate, and 85.7% after 12 and 24 h, and 90.5% after 7 days for n-butanol, while the n-hexane extract was ineffective [7, 109].

Among the potent identified bioactive secondary metabolites, the volatile phenolic compounds 2,4-ditert-butylphenol and 3,5-ditert-butylphenol displayed significant toxicity against a wide range of organisms, demonstrating antimicrobial, insecticidal, and nematicidal activities [110, 111]. The fatty acid derivatives, including butyl 13-methyltetradecanoate, 2-hydroxy-1-[(palmitoyloxy)methyl]ethyl palmitate, and others, produced by marine *B. gottheilii* MSB1, have been reported to exhibit antibacterial and antifungal properties [112, 113]. Other bioactive compounds such as 4-amino-6-hydroxy-2-methyl-5-nitrosopyrimidine, 3,3-dimethyl-6-(methylsulfanyl)-8-(4-morpholinyl)-3,4-dihydro-1 H-thiopyrano[3,4-c]pyridine-5-carbonitrile, and others demonstrated mild anxiolytic, antifungal, antibacterial, antioxidant, and antimalarial activities [114, 115].

The modes of action of many antimicrobials are complex and may affect multiple targets. The phenomenon of membrane bleeding has been observed with several antimicrobial agents [116]. For instance, phenolic and flavonoid compounds exert their effects through various mechanisms, including membrane disruption [117], protein binding, inhibition of protein synthesis, enzyme inhibition, and production of cell wall complexes [118]. Alkaloids may inhibit critical enzymes or act as DNA-intercalating agents [119]. Lysozymes affect bacterial cell walls and membranes, leading to membrane disruption, release of intracellular contents, and subsequent bacterial cell death [120].

Rongai et al. [121]. reported that certain plant groups, including Asteraceae, Oleaceae, and Lamiaceae, exhibit fungicidal action against *Alternaria* and *Fusarium* species. Chandel and Kumar also confirmed that the extract from *Calliandra callothyrsus* inhibits the growth of *Alternaria alternata*, *Alternaria solani*, *Phoma* sp., *Fusarium* sp., and *Aspergillus* sp., and promotes pea germination.

According to Daradka et al. [122], plants analyzed belonged to several families. Three dosages of plant extracts (10, 50, and 100 mg/ml) were tested against *A. alternata* and *F. oxysporum* by determining the inhibition zone of fungal mycelial growth using the disc-diffusion method on Potato Dextrose Agar (PDA), yielding significant results. The antifungal activity of plant extracts (50–100 mg/mL) against *A. alternata* and *F. solani* was assessed by monitoring mycelium radial growth and calculating the minimum inhibitory concentration (MIC) according to Lira-De et al. [123].

In the present study, we report the first isolation of *B.gottheilii* MSB1 from a marine sponge. Previously, this bacterium was isolated from mangrove sediment in Peninsular Malaysia [124]. *B. gottheilii* MSB1 is a Gram-positive, rod-shaped, motile, strictly aerobic, endospore-forming bacterium with potential applications in pharmaceutical production [125]. We investigated the antimicrobial and nematicidal activities of *B. gottheilii* MSB1 extracts in vitro, as well as its biocontrol efficacy against *Fusarium oxysporum*, *Alternaria alternata*, and *Meloidogyne incognita* under laboratory conditions. The results revealed that the extracts exhibit significant antimicrobial and nematicidal effects, which are consistent with previous studies [29, 126].

In considering the promising antimicrobial and nematicidal properties of *B. gottheilii* MSB1, it is essential to explore the broader implications of these findings, particularly in sustainable agriculture and pest management. The potential reduction in chemical pesticide use offers significant environmental and health benefits, aligning with global efforts to reduce chemical footprints in agricultural practices. However, the pathway to practical application is not without challenges. Implementing these biocontrol strategies on a large scale requires a thorough analysis of economic feasibility, logistical considerations, and farmer adoption hurdles. Factors such as production costs, delivery mechanisms, compatibility with existing agricultural systems, and regulatory approvals must be addressed to ensure that these biological solutions are not only effective but also accessible and attractive to farmers worldwide. Furthermore, understanding the long-term impact on ecosystems and crop yield stability will be crucial to guarantee that such innovations contribute positively to sustainable agricultural landscapes [127–130]. By engaging in a comprehensive evaluation of these elements, stakeholders can better facilitate the integration of *B. gottheilii* MSB1 into real-world agricultural settings, thereby maximizing its potential benefits .

This study demonstrates the potential of *B. gottheilii* MSB1, isolated from the marine sponge Hyrtios erecta, as a source of bioactive secondary metabolites with significant antimicrobial and nematicidal properties. These findings support the viability of marine microorganisms as eco-friendly alternatives for managing plant pathogens, reducing agrochemical use, and enhancing sustainable agriculture. The identified bioactive compounds, such as volatile phenolic compounds and fatty acid derivatives, show promising applications in agriculture and pharmaceuticals.

Nonetheless, the study has limitations; the activity of *B. gottheilii* MSB1 could vary with environmental conditions, which were not fully examined here. Furthermore, while several bioactive compounds were identified, the specific mechanisms by which they disrupt fungal or bacterial growth remain unclear. Future research should focus on elucidating these mechanisms to maximize the scientific contribution and potential applications of these compounds. Challenges such as large-scale production and purification also need addressing to facilitate practical use, ensuring *B. gottheilii* MSB1’s efficacy as a biocontrol agent across different environments.

## Conclusions

This study confirms that Bacillus species, including *B. gottheilii* MSB1, exhibit potential as agents against plant pathogens, such as fungi and nematodes, suggesting their possible role as producers of antibacterial, antifungal, nematicidal, and biocontrol agents in agricultural contexts. While these findings highlight the potential of Bacillus species in integrated pest management strategies, it is important to acknowledge that the current research primarily reflects laboratory results. The translation of these findings to real-world agricultural applications requires further steps, including comprehensive field trials and toxicity assessments, to validate their efficacy and safety on a large scale. Additionally, the study does not yet explore how these applications may directly contribute to environmental sustainability and the United Nations’ Sustainable Development Goals (SDGs). Therefore, while promising, further research is essential to establish the practical, sustainable use of *B. gottheilii* MSB1 extracts in agriculture.

## Electronic supplementary material

Below is the link to the electronic supplementary material.

Supplementary Material 1

## Data Availability

All data generated or analyzed during this study are included in this manuscript and its information files and supplementary file.

## Abbreviations

QRT-PCR: Quantitative Real-Time PCR
*PR2*: β-1,3-glucanases
*PR3*: Pathogenesis-related protein 3
*PR4*: Pathogenesis-related protein 4
*PR5*: Thaumatin-like proteins
EF: Elongation factor
VOC: Volatile Organic Compounds
VOCs: Volatile Organic Compounds (plural)
*A. alternata*: Alternaria alternata
*F. oxysporum*: Fusarium oxysporum
