# Supplementary material for: Isolation and identification of antifungal, antibacterial and nematocide agents from marine bacillus gottheilii MSB1
Source: BMC Biotechnol. 2024 Nov 13;24:92. doi: 10.1186/s12896-024-00920-y (PMC11562594; doi:10.1186/s12896-024-00920-y)
Supplement: Supplementary file 1 — Supplementary Material 1 [file 12896_2024_920_MOESM1_ESM.pdf]

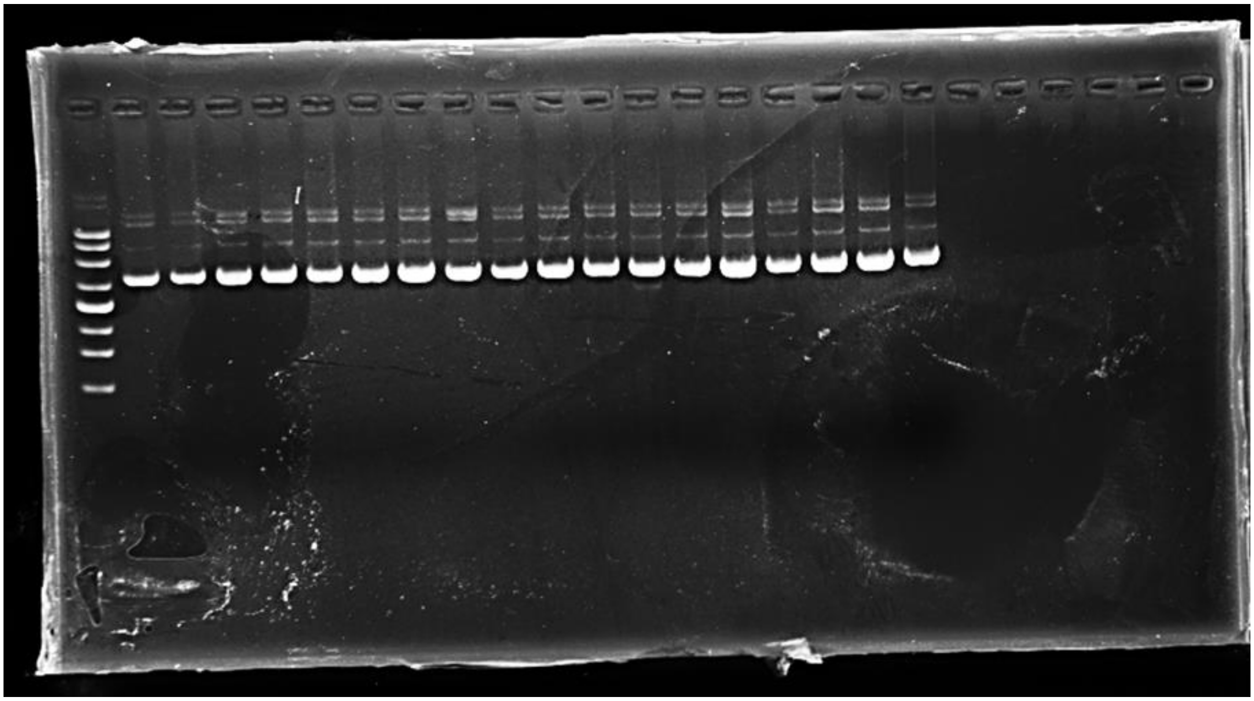

Fig. S1. Full uncropped gel showing the amplified region of the Bacillus sp. isolate-specific genes, with an approximate size of 1369 bp.
